# Bayesian optimization of Fisher Information in nonlinear multiresonant quantum photonics gyroscopes

**DOI:** 10.1515/nanoph-2024-0032

**Published:** 2024-03-22

**Authors:** Mengdi Sun, Vassilios Kovanis, Marko Lončar, Zin Lin

**Affiliations:** Bradley Department of Electrical and Computer Engineering, 1757 Virginia Tech, Arlington, VA, USA; John A. Paulson School of Engineering and Applied Sciences, Harvard University, Cambridge, MA, USA; Bradley Department of Electrical and Computer Engineering, 1757 Virginia Tech, Blacksburg, VA, USA

**Keywords:** optical gyroscope, nonlinear optics, quantum photonics, Bayesian optimization, Fisher Information

## Abstract

We propose an on-chip gyroscope based on nonlinear multiresonant optics in a thin film *χ* ^(2)^ resonator that combines high sensitivity, compact form factor, and low power consumption simultaneously. We theoretically analyze a novel *holistic* metric – Fisher Information capacity of a multiresonant nonlinear photonic cavity – to fully characterize the sensitivity of our gyroscope under fundamental quantum noise conditions. Leveraging Bayesian optimization techniques, we directly maximize the nonlinear multiresonant Fisher Information. Our *holistic* optimization approach orchestrates a harmonious convergence of multiple physical phenomena – including noise squeezing, nonlinear wave mixing, nonlinear critical coupling, and noninertial signals – all encapsulated within a single sensor-resonator, thereby significantly augmenting sensitivity. We show that ∼470× improvement is possible over the shot-noise limited linear gyroscope with the same footprint, intrinsic quality factors, and power budget.

## 1 Introduction

Gyroscopes are critical components of an inertial navigation system for augmenting the GPS guidance or salvaging GPS-denied operational environments [1]. In an optical gyroscope, the rotation rate is measured through the phase shift between two counter-propagating beams in an optical loop. This approach was first proposed by Sagnac in 1913 [2], [3] and soon different types of optical gyroscopes were developed [4], [5]. Careful studies have been performed on the sensitivity and the quantum noise of these gyroscopes [6], [7], and remarkable levels of sensitivity ( <0.001° /h) have been achieved in state-of-the-art discrete component optical gyroscopes, including fiber optic gyroscopes (FOG) [8], [9], [10], ring laser gyroscopes (RLG) [11], atom-laser gyroscope [12], and optical cavity gyroscopes [13], [14], [15]. However, bulky components and relatively high power consumption remain major roadblocks to further exploiting discrete component optical gyroscopes. On the other hand, on-chip optical gyroscopes [16], [17], [18], [19], [20] exhibit great potential for fully integrated inertial navigation platforms (free of fragile moving parts) and can outperform their discrete component counterparts in size, weight, power consumption, maneuverability, manufacturing scalability, robustness, and the ability to operate in harsh environments. However, on-chip gyros are yet to reach sensitivity levels smaller than 1°/h. This is due to fundamentally limited optical path lengths even in ultra-high quality factor resonators [16], leaving dubious prospects for further improvements via increasing resonator size or quality factors. To address the challenge of this seemingly intrinsic trade-off between sensitivity and compactness, novel physics and designs have been investigated, including exceptional point sensing [21], [22], slow light [23], [24], dispersive enhancements [25], [26], dynamic thermal drift cancellation [15], [27], and nuclear magnetic resonance [28].

Meanwhile, driven by the emerging trend of quantum technologies [29], [30], [31], quantum light sensors have been identified as a *promising* option that can extend the fundamental sensitivity limits beyond the shot noise regime [32], [33], [34]. These ideas were reinforced by decades of development and analysis that lead to the construction of very large laser interferometers with extreme sensitivity that is capable of detecting gravitational waves from remote cosmological events. Recently, squeezed light was used in the LIGO in the US and the VIRGO in Italy to substantially improve the sensitivity of the observing runs that happened late in April 2019 [35], [36]. On the other hand, recent advances in nanofabrication, integration, and packaging of ultra-coherent laser sources [37], low-loss photonic circuits [38], [39], and highly efficient photo-detectors [40] have opened up exciting opportunities for realizing fully on-chip quantum devices. Along this trend, we identify on-chip optical gyroscopes, operating under fundamental quantum noise conditions, as promising candidates for next-generation rotation sensing.

In this paper, we theoretically introduce a new type of on-chip nonlinear multiresonant gyroscope in integrated thin film resonators with strong quadratic *χ* ^(2)^ nonlinearities that simultaneously achieve high sensitivity, high compactness, and low power consumption. Instead of externally injecting squeezed states of light into the gyroscope [33], [41], our gyroscope design *fuses* nonlinear wave mixing, noise squeezing and cancellations, and noninertial signal accumulation inside the same sensor-resonator, enabling ≳470× improvements in gyroscopic sensitivity over the linear shot noise limit. In our scheme, classical laser light (coherent state) is injected into a doubly resonant *χ* ^(2)^ cavity, and output light is measured at the fundamental (*ω*_1_) and second harmonic frequencies (*ω*_2_ = 2*ω*_1_). The sensitivity of the gyroscope is evaluated by Fisher Information (FI) [42], [43], and the latter is maximized by Bayesian optimization [44]. Various parameter regimes associated with both fundamental and second harmonic injection schemes were investigated, which reveal correlated noise suppression and sensitivity enhancements via parametric oscillations and critically sensitive three wave mixing dynamics. We predict that, under quantum noise conditions, a minimum detectable rotation rate (MDR) of <0.01° /h can be achieved using a thin film lithium niobate (TFLN) ring resonator with a diameter of 20 mm, intrinsic quality factors *Q*_*i*2_ = 10^6^ at the second harmonic wavelength (795 nm), *Q*_*i*1_ = 10^7^ at the fundamental wavelength (1590 nm). We discuss the scope, validity, and implications of our approach and results, while the key sensitivity enhancement factors are summarized in Table 1 of Section 4.

**Table 1:** Optimal sensitivities of various injection schemes.

	*P*_1_ (mW)	*P*_2_ (m*W*)	*Q*_*c*1_	*Q*_*c*2_	Ω_min_ (°/h)
Linear	23.507	None	9.58 × 10^6^	None	0.278
**SHI**	None	23.507	1.018 × 10^5^	5.462 × 10^5^	**0.004465**
**Sensitivity enhancement factor: 62.3×**					
Linear	0.000945	None	9.58 × 10^6^	None	43.81
**FFI**	None	0.000945	6.747 × 10^6^	6.765 × 10^7^	**0.09296**
**Sensitivity enhancement factor: 471.3×**					

The bold values represent the optimal sensitivities and the corresponding enhancement factors at each injection scheme.

## 2 Gyroscopic model

### 2.1 Linear resonant gyroscope as a baseline

**Figure 1:**
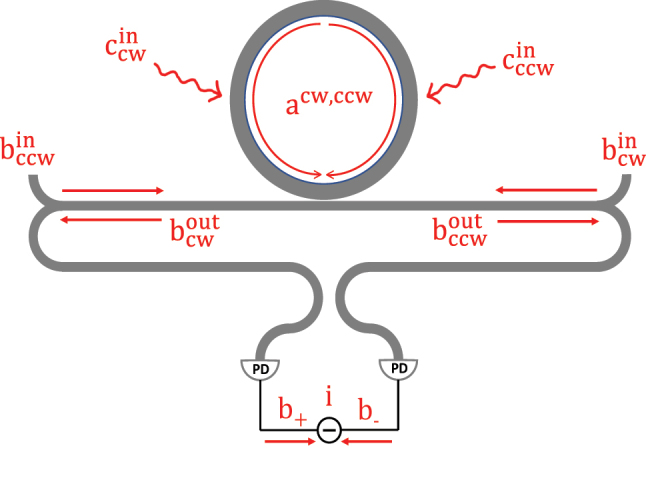
The schematic of the linear micro-ring gyroscope. The input and output light of the micro-ring cavity is injected at two waveguide ports $b_{1}^{\text{in}}$ (CW)/ $b_{1}^{\text{in}}$ (CCW) and $b_{1}^{\text{out}}$ (CW)/ $b_{1}^{\text{out}}$ (CCW). The radiation losses are expressed by the fictitious radiation channel $c_{1}^{\text{in}}$ (CW)/ $c_{1}^{\text{in}}$ (CCW). The output light is measured by homodyne detection.

We first review a basic interferometric scheme probing the gyroscopic shift of a linear resonant cavity, as outlined in Figure 1. We perform a quantum noise analysis similar to Ref. [42] or Section 4 of Ref. [7]. Two identical counter-propagating probes (seeded from the same on-chip laser) are injected into the clockwise (CW) and the counterclockwise (CCW) modes of a ring resonator; at the exit, the two probe fields are set to interfere via balanced homodyne detection [45]. In the absence of rotation, the CW and the CCW modes are degenerate and the exiting fields register a vanishing *differential* photocurrent signal at the detection setup [7]. Rotational motion induces a frequency splitting proportional to the rotation rate Ω, which in turn induces a phase difference between the outgoing CW and CCW probes. Subsequently, interference of the two probe fields gives rise to a nonzero differential signal and the underlying Ω can be measured. For conceptual simplicity, we assume that the frequency of the probe laser is always locked to the degenerate frequency of the unperturbed gyro [7]. In principle, this can be achieved by self-injection locking the laser to an independent rotation-insensitive cavity (such as a high-Q spiral resonator [46]) having the exact same frequency as the unperturbed gyro ring. It has been demonstrated [47] that self-injection locking to a high-Q cavity can produce an ultra-coherent *integrated* laser with a sub-Hertz linewidth; therefore, we can readily approximate the laser state as a coherent state. In the Heisenberg picture, the Hamiltonian of a linear optical gyroscope is given by:

(1) $$\begin{array}{ll} H & =\sum \hbar \omega {\hat{a}}^{†}\hat{a}+\sum i\hbar \frac{\kappa }{2}\left({\hat{b}}^{†}\hat{a}+{\hat{a}}^{†}\hat{b}\right)\\ & \;+\sum \hbar \beta \left({\hat{a}}_{\text{cw}}^{†}{\hat{a}}_{\text{ccw}}+{\hat{a}}_{\text{ccw}}^{†}{\hat{a}}_{\text{cw}}\right) \end{array}$$

Then, we can derive the Heisenberg–Langevin equations that the ring resonator gyro obeys [48]:

(2) $$\frac{d{\hat{a}}_{\text{cw}}}{dt}=\left(-\frac{\kappa }{2}-\frac{\gamma }{2}+i\delta \right){\hat{a}}_{\text{cw}}+i\beta {\hat{a}}_{\text{ccw}}+\sqrt{\kappa }{\hat{b}}_{\text{cw}}^{\text{in}}+\sqrt{\gamma }{\hat{c}}_{\text{cw}}^{\text{in}}$$

(3) $$\frac{d{\hat{a}}_{\text{ccw}}}{dt}=\left(-\frac{\kappa }{2}-\frac{\gamma }{2}-i\delta \right){\hat{a}}_{\text{ccw}}+i\beta {\hat{a}}_{\text{cw}}+\sqrt{\kappa }{\hat{b}}_{\text{ccw}}^{\text{in}}+\sqrt{\gamma }{\hat{c}}_{\text{ccw}}^{\text{in}}$$

where ${\hat{a}}_{\text{cw}}$ and ${\hat{a}}_{\text{ccw}}$ are the annihilation operators for the cavity CW and CCW modes excited by the injections ${\hat{b}}_{\text{cw}}^{\text{in}}$ and ${\hat{b}}_{\text{ccw}}^{\text{in}}$ , respectively. ${\hat{c}}_{\text{cw}}^{\text{in}}$ and ${\hat{c}}_{\text{ccw}}^{\text{in}}$ represent intrinsic loss channels (such as radiative losses). *κ* and *γ* are the decay rates for the coupling and the intrinsic losses. We approximate Rayleigh-type back-scattering as a linear (conservative) coupling *β* between CW and CCW modes inside the cavity [16]. *δ* is the rotation-induced resonant frequency shift due to the Sagnac effect. Here, we have assumed single-photon normalization for each eigenmode so that ${\hat{a}}^{†}\hat{a}$ , for example, represents the photon number operator inside the cavity.

We denote $\left\langle \hat{A}\right\rangle =〈\psi |\hat{A}|\psi 〉$ as the usual notation for computing the expectation value of a physical observable $\hat{A}$ with respect to the quantum state |*ψ*⟩. In the linear problem, we will consider coherent states of the same amplitude *b* ^in^ in the input waveguides and vacuum states in the intrinsic loss channels for both CW and CCW light [7]. The input state of the gyro is then given by $|\psi 〉={|b^{\text{in}}〉}_{\text{cw}}{|b^{\text{in}}〉}_{\text{ccw }}|{0〉}_{\text{cw}}|{0〉}_{\text{ccw}}$ . The classical counterpart of the input operator ${\hat{b}}^{\text{in}}$ is the input amplitude of a coherent state in the feeder waveguide and can be related to the input power *P* by the formula:

(4) $$|b^{\text{in}}{|}^{2}=\left\langle {\hat{b}}^{\text{in}†}{\hat{b}}^{\text{in}}\right\rangle =\frac{P}{\hbar \omega }$$

The output operators in the waveguides are given by [7]:

(5) $${\hat{b}}_{\text{cw}}^{\text{out}}={\hat{b}}_{\text{cw}}^{\text{in}}-\sqrt{\kappa_{1}}{\hat{a}}_{\text{cw}}$$

(6) $${\hat{b}}_{\text{ccw}}^{\text{out}}={\hat{b}}_{\text{ccw}}^{\text{in}}-\sqrt{\kappa_{1}}{\hat{a}}_{\text{ccw}}$$

The clockwise and counterclockwise signals are set to interfere through a directional coupler/beam splitter with a controllable phase shift *ϕ*, followed by photodetection. The signal incident on the photodetectors and the photocurrent operators are then given by:

(7) $${\hat{b}}_{+}=\left({\hat{b}}_{\text{cw}}^{\text{out}}{\text{e}}^{\text{i}\phi /2}+i{\hat{b}}_{\text{ccw}}^{\text{out}}{\text{e}}^{-\text{i}\phi /2}\right)/\sqrt{2}$$

(8) $${\hat{b}}_{-}=\left(i{\hat{b}}_{\text{cw}}^{\text{out}}{\text{e}}^{\text{i}\phi /2}+{\hat{b}}_{\text{ccw}}^{\text{out}}{\text{e}}^{-\text{i}\phi /2}\right)/\sqrt{2}$$

(9) $${\hat{i}}_{+}={\hat{b}}_{+}^{†}{\hat{b}}_{+}$$

(10) $${\hat{i}}_{-}={\hat{b}}_{-}^{†}{\hat{b}}_{-}$$

We measure the differential current signal:

(11) $$\hat{i}={\hat{i}}_{+}-{\hat{i}}_{-}$$

As a figure of merit, we will investigate the minimum detectable frequency shift by calculating the ratio between the standard variation of the measured differential current and the derivative of the mean value of the current over the rotation-induced frequency shift, as reported by Dowling in 1998 [12]:

(12) $$\delta_{\min}={\left.\frac{\sqrt{\left\langle {\hat{i}}^{2}\right\rangle -{\left\langle \hat{i}\right\rangle }^{2}}}{\left|\frac{\partial \left\langle \hat{i}\right\rangle }{\partial \delta }\right|}\right|}_{\delta =0}$$

Since the resonant frequency shift due to the Sagnac effect is given by $\delta =\frac{2\pi r\Omega }{\lambda n_{0}}$ [7], the minimum detectable rotation rate (MDR) is given by:

(13) $$\Omega_{\min}={\left.\frac{\lambda n_{0}}{2\pi R}\frac{\sqrt{\left\langle {\hat{i}}^{2}\right\rangle -{\left\langle \hat{i}\right\rangle }^{2}}}{\left|\frac{\partial \left\langle \hat{i}\right\rangle }{\partial \delta }\right|}\right|}_{\delta =0}$$

where *R* and *n*_0_ are the radius and the refractive index of the micro-ring. *λ* is the wavelength of the input light. We emphasize that Ω_min_ is a holistic measure, which not only considers the sensitivity of the noise-averaged photocurrent with respect to Ω but also the variance of the measured current signals *due to noise* (both are critical to correctly characterizing the overall performance of the gyro; it has been pointed out [49] that an analysis only of the photocurrent sensitivity could often lead to misleading conclusions).

If we ignore Rayleigh back-scattering *β* = 0, we can derive a simple closed-form expression for MDR in the linear gyroscope (LG):

(14) $$\Omega_{\min}^{\text{LG}}=\frac{\sqrt{2}\lambda n_{0}{\left(\kappa +\gamma \right)}^{2}}{64\pi R\kappa \sqrt{N}}$$

where $N=\frac{P}{\hbar \omega }$ is the incident number of photons per unit time. $\Omega_{\min}^{\text{LG}}$ is minimized at *κ* = *γ*, yielding ${\text{MDR}}_{\min}^{\text{LG}}=\frac{\sqrt{2}cn_{0}}{8R\sqrt{N}Q_{i}}$ , where the intrinsic quality factor is defined by $Q_{i}=\frac{\omega }{\gamma }$ . Note that Eq. (14) is only a simplification to illustrate the simple sensitivity dependence of a linear gyroscope in limit of vanishing back-scattering. We will fully take into account back-scattering effects in our ensuing works. We note that, in our analysis, we only consider fundamental quantum noise: without loss of generality, we have assumed perfect beam splitters and detectors external to the resonator, while we do consider realistic losses inside the resonator. This linear result will serve as a baseline comparison for our later analysis of a new mode of gyroscope that relies on nonlinear interactions. Note that the scaling ${\text{MDR}}_{\min}^{\text{LG}}∼\frac{1}{\sqrt{N}Q_{i}}$ recovers the familiar shot noise limit [12]. In addition, the $\frac{1}{R}$ dependence in Eq. (14) indicates that a larger ring radius *R* offers better sensitivity, which is one of the common control knobs of classical linear optical gyroscope.

### 2.2 Nonlinear multiresonant quantum photonics gyro

**Figure 2:**
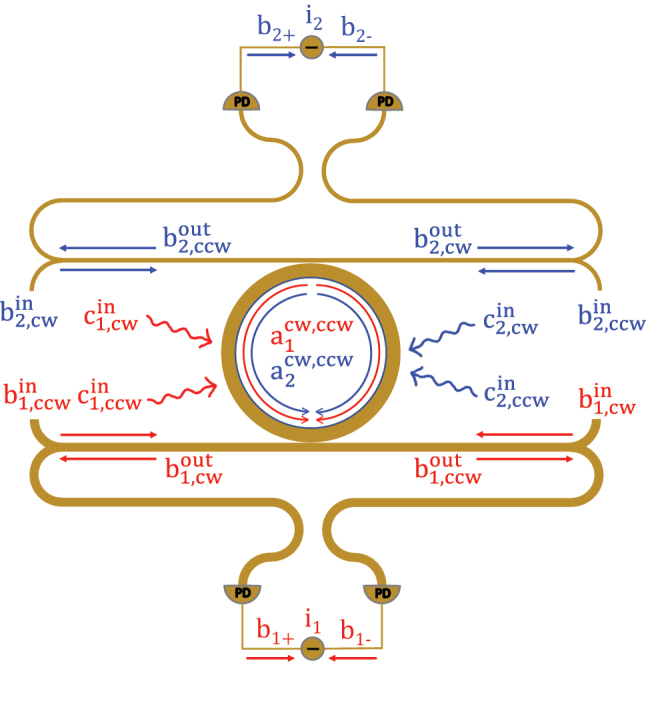
The schematic of the nonlinear micro-ring gyroscope. The input light is injected at four waveguide ports $b_{1}^{\text{in}}$ (CW)/ $b_{1}^{\text{in}}$ (CCW) and $b_{2}^{\text{in}}$ (CW)/ $b_{2}^{\text{in}}$ (CCW). The input/output and loss channels for the second harmonic light are expressed by blue arrows. The output light is measured by a joint measurement using the differential currents at both output ports ( $b_{1}^{\text{out}}$ and $b_{2}^{\text{out}}$ ). The accuracy of the measurement is determined by evaluating the Fisher Information of the output light.

We now consider the gyroscopic operation of a doubly resonant ring resonator with quadratic *χ* ^(2)^ nonlinearities (Figure 2). Quadratic nonlinearities are well-known generators of correlations such as squeezing and entanglement [50], [51]. Our nonlinear multiresonant gyro synergistically leverages nonlinear dynamics, noise squeezing and cancellations, and noninertial Sagnac effects in the same sensor-resonator, to improve the gyroscopic sensitivity. Specifically, based on the Hamiltonian of the nonlinear gyroscope:

(15) H=∑ℏωi,ja^i,j†a^i,j+∑iℏκi2b^i,j†a^i,j+a^i,j†b^i,j+∑iℏχ2a^1,j†a^2,j2−a^1,j2a^2,j†+∑ℏβia^i,cw†a^i,ccw+a^i,ccw†a^i,cw

we investigate the following Heisenberg–Langevin equations:

(16) $$\begin{array}{ll} \frac{d{\hat{a}}_{1,\text{cw}}}{dt} & =-\left(\frac{\kappa_{1}}{2}+\frac{\gamma_{1}}{2}-i\delta_{1}\right){\hat{a}}_{1,\text{cw}}+i\beta_{1}{\hat{a}}_{1,\text{ccw}}\\ & \;+\chi {\hat{a}}_{1,\text{cw}}^{†}{\hat{a}}_{2,\text{cw}}+\sqrt{\kappa_{1}}{\hat{b}}_{1,\text{cw}}^{\text{in}}+\sqrt{\gamma_{1}}{\hat{c}}_{1,\text{cw}}^{\text{in}} \end{array}$$

(17) $$\begin{array}{ll} \frac{d{\hat{a}}_{1,\text{ccw}}}{dt} & =-\left(\frac{\kappa_{1}}{2}+\frac{\gamma_{1}}{2}+i\delta_{1}\right){\hat{a}}_{1,\text{ccw}}+i\beta_{1}{\hat{a}}_{1,\text{cw}}\\ & \;+\chi {\hat{a}}_{1,\text{ccw}}^{†}{\hat{a}}_{2,\text{ccw}}+\sqrt{\kappa_{1}}{\hat{b}}_{1,\text{ccw}}^{\text{in}}+\sqrt{\gamma_{1}}{\hat{c}}_{1,\text{ccw}}^{\text{in}} \end{array}$$

(18) $$\begin{array}{ll} \frac{d{\hat{a}}_{2,\text{cw}}}{dt} & =-\left(\frac{\kappa_{2}}{2}+\frac{\gamma_{2}}{2}-i\delta_{2}\right){\hat{a}}_{2,\text{cw}}+i\beta_{2}{\hat{a}}_{2,\text{ccw}}\\ & \;-\frac{1}{2}\chi {\hat{a}}_{1,\text{cw}}^{2}+\sqrt{\kappa_{2}}{\hat{b}}_{2,\text{cw}}^{\text{in}}+\sqrt{\gamma_{2}}{\hat{c}}_{2,\text{cw}}^{\text{in}} \end{array}$$

(19) $$\begin{array}{ll} \frac{d{\hat{a}}_{2,\text{ccw}}}{dt} & =-\left(\frac{\kappa_{2}}{2}+\frac{\gamma_{2}}{2}+i\delta_{2}\right){\hat{a}}_{2,\text{ccw}}+i\beta_{2}{\hat{a}}_{2,\text{cw}}\\ & \;-\frac{1}{2}\chi {\hat{a}}_{1,\text{ccw}}^{2}+\sqrt{\kappa_{2}}{\hat{b}}_{2,\text{ccw}}^{\text{in}}+\sqrt{\gamma_{2}}{\hat{c}}_{2,\text{ccw}}^{\text{in}} \end{array}$$

In these equations, the index *j* = 1, 2 in the field operators ( ${\hat{a}}_{j}$ , ${\hat{b}}_{j}$ , ${\hat{c}}_{j}$ ) stands for the fundamental *ω*_1_ and second harmonic *ω*_2_ = 2*ω*_1_ resonances. *κ*_*j*_ and *γ*_*j*_ are the decay rates of the coupling and the intrinsic loss channels. Rayleigh scattering rate between CW and CCW modes at each resonance *j* is again characterized by *β*_*j*_, which is inversely proportional to the intrinsic quality factor at each resonance *j* (*Q*_*ij*_). The nonlinear coupling terms $\chi {\hat{a}}_{1}^{†}{\hat{a}}_{2}$ indicate a multi-photon process in which one incident photon with *ω*_2_ breaks down into two photons of half the frequency $\omega_{1}=\frac{\omega_{2}}{2}$ (parametric down conversion [52]), or its reverse, $\frac{1}{2}\chi {\hat{a}}_{1}^{2}$ , indicating that two photons with *ω*_1_ combine into one photon with the double frequency *ω*_2_ = 2*ω*_1_ (second harmonic generation [53]). These are energy-conserving, three-wave mixing processes, which preserve the fundamental commutation relations [54]. The rotation-induced frequency shifts (*δ*_1_, *δ*_2_) are different for each resonance, have opposite polarity between CW and CCW modes, and can be approximated by *δ*_2_ = 2*δ*_1_ (since $\delta =\frac{2\pi r\Omega }{\lambda n_{0}}$ [7]). Note that here the material dispersion of the lithium niobate is neglected because the index difference (*n* = 2.21 at 1590 nm and *n* = 2.25 at 795 nm) can be compensated by engineering the geometry dispersion [55]. In order to improve the accuracy of the model, however, the dispersion effect should be taken into account in future exploration. The key parameter in this model is the nonlinear modal coupling strength [48], [56]:

(20) $$\chi =\frac{\epsilon_{0}}{\hbar }∭\frac{3\chi^{\left(2\right)}\left(r\right)}{4\sqrt{2}}u_{1}^{*}{\left(z,r,\theta \right)}^{2}u_{2}\left(z,r,\theta \right)rdrd\theta dz$$

where $u_{1}^{*}\left(z,r,\theta \right)$ and *u*_2_(*z*, *r*, *θ*) are the electric field profiles (in polar coordinates) of the fundamental and the second harmonic eigenmodes of the gyroscopic resonator. Given that our sensor is a ring resonator of radius *R*, it is instructive to decompose *χ* into cross-sectional modal overlap *ζ* and the remaining contributions. Following [57], we approximate:

(21) $$\chi \approx \sqrt{\frac{\hbar \omega_{1}^{2}\omega_{2}}{\epsilon_{0}2\pi R}}\frac{\zeta }{\epsilon_{1}\sqrt{\epsilon_{2}}}\frac{3\chi^{\left(2\right)}}{4\sqrt{2}}$$

(22) $$\zeta =\frac{\iint u_{1}^{*}{\left(z,r\right)}^{2}u_{2}\left(z,r\right)drdz}{\iint |u_{1}^{*}\left(z,r\right){|}^{2}drdz\sqrt{\iint |u_{2}\left(z,r\right){|}^{2}drdz}}$$

It is important to realize that the nonlinear Langevin equations [48], [58] encode the time evolution of *four coupled infinite-dimensional* quantum operators; as such, it is very challenging to obtain an exact solution either analytically or numerically (we note that straightforward numerical methods using a truncated Fock basis [59] are not feasible because our system typically involves milli-watts of optical power amounting to $∼10^{16}$ photons). However, at milli-watt injection powers, quantum fluctuations can be considered “small signals” compared to much stronger average field intensities at steady state, so that each operator can be decomposed into a classical scalar-valued amplitude and a fluctuation operator, e.g., $\hat{a}=\alpha +\delta \hat{a}$ . The details of calculating steady state solutions are included in the Supplementary Material 1. The classical amplitude represents a steady-state solution to the mean-field averaged Langevin equations at the classical (large photon number) limit while the “small-signal” fluctuation operator approximately obeys the linearized Langevin equations in the vicinity of the steady-state mean-field solution. Linearizing a nonlinear steady state to study the fluctuations in its vicinity is commonly known as small-signal modeling in electronics engineering [60]. In a similar spirit, the small-signal treatment of the fluctuation operators in the Heisenberg–Langevin picture is a simple but effective approach widely accepted for steady-state noise analysis in laser and nonlinear quantum optics literature with experimental support [7], [61], [62], [63], [64]. Theoretically, it is important to note that such an approach is justified as long as the steady state we consider is a *stable* hyperbolic fixed point to which all nearby trajectories converge, ensuring small fluctuations (Hartman–Grobman theorem [65]). On the other hand, a more sophisticated phase-space formalism, which employs quasi-probability distributions, Fokker–Planck equations, and stochastic calculus, can be used to study more complicated dynamics such as large fluctuations at nonhyperbolic critical points and self-pulsing (limit-cycle) solutions [66]. Using the small-signal approximation, we can compute the differential photocurrent signals at both the fundamental and the second harmonic resonances (see also Figure 2):

(23) $${\hat{i}}_{1}=iA_{1}\left({\hat{b}}_{1\text{,cw}}^{\text{out}†}{\hat{b}}_{1\text{,ccw}}^{\text{out}}{\text{e}}^{-\text{i}\phi_{1}}-{\hat{b}}_{1\text{,ccw}}^{\text{out}†}{\hat{b}}_{1\text{,cw}}^{\text{out}}{\text{e}}^{\text{i}\phi_{1}}\right)$$

(24) $${\hat{i}}_{2}=iA_{2}\left({\hat{b}}_{2\text{,cw}}^{\text{out}†}{\hat{b}}_{2\text{,ccw}}^{\text{out}}{\text{e}}^{-\text{i}\phi_{2}}-{\hat{b}}_{2\text{,ccw}}^{\text{out}†}{\hat{b}}_{2\text{,cw}}^{\text{out}}{\text{e}}^{\text{i}\phi_{2}}\right)$$

Here, *A*_1_ and *A*_2_ are constant factors determined by the frequencies of the light and the responsivity of the photodetectors. ${\text{e}}^{\text{i}\phi_{1}}$ and ${\text{e}}^{\text{i}\phi_{2}}$ are the propagation phase shifts that each output light experiences, which are determined by the propagation distances after the output CW and CCW waves are mixed. Therefore, by controlling the lengths of the output waveguides, ${\text{e}}^{\text{i}\phi_{1}}$ and ${\text{e}}^{\text{i}\phi_{2}}$ are set to one here. Here, we measure both ${\hat{i}}_{1}$ and ${\hat{i}}_{2}$ to extract maximal information out of the nonlinear wave-mixing gyro.

The output of our nonlinear multiresonant gyroscope is now characterized by a mean vector ⟨**i**⟩ and a covariance matrix **Σ**:

(25) $$\left\langle i\right\rangle =\left(\begin{array}{c} \left\langle {\hat{i}}_{1}\right\rangle \\ \left\langle {\hat{i}}_{2}\right\rangle \end{array}\right)$$

(26) $$\Sigma =\left(\begin{array}{cc} \left\langle {\hat{i}}_{1}^{2}\right\rangle -{\left\langle {\hat{i}}_{1}\right\rangle }^{2} & \frac{\left\langle {\hat{i}}_{1}{\hat{i}}_{2}\right\rangle +\left\langle {\hat{i}}_{2}{\hat{i}}_{1}\right\rangle }{2}-\left\langle {\hat{i}}_{1}\right\rangle \left\langle {\hat{i}}_{2}\right\rangle \\ \frac{\left\langle {\hat{i}}_{1}{\hat{i}}_{2}\right\rangle +\left\langle {\hat{i}}_{2}{\hat{i}}_{1}\right\rangle }{2}-\left\langle {\hat{i}}_{2}\right\rangle \left\langle {\hat{i}}_{1}\right\rangle & \left\langle {\hat{i}}_{2}^{2}\right\rangle -{\left\langle {\hat{i}}_{2}\right\rangle }^{2} \end{array}\right)$$

Assuming that the joint probability distribution of the measured photocurrents follow a bivariate Gaussian, we can express the Fisher Information [43] of our gyroscope:

(27) $$I\left(\delta \right)={\left(\frac{d〈i〉}{d\delta }\right)}^{T}\Sigma^{-1}\left(\frac{d〈i〉}{d\delta }\right)$$

The details of the statistical analysis of the differential currents are included in Supplementary Material 2. Then the sensitivity is determined by Cramer–Rao bound [67]:

(28) $$\delta_{\min}=\frac{1}{\sqrt{I\left(\delta \right)}}$$

Similar to the linear gyroscope, here MDR is given by:

(29) $$\Omega_{\min}=\frac{\lambda n_{0}}{2\pi R}\delta_{\min}$$

Before we provide further estimation for particular gyroscope implementation, we want to offer a few remarks on our modeling approach:

– In this paper, we have stuck to a Langevin description of our nonlinear gyroscope, which takes into account quantum noise through the Langevin fluctuation operator $\hat{b}$ or $\hat{c}$ in each coupling or dissipation channel (with the rates determined by the fluctuation–dissipation theorem), preserving the fundamental commutation relations [48].
  We note that while the Langevin form is widely utilized in many experimental situations [57], more sophisticated theoretical analysis, delineating the open-system quantum dynamics [68], can be performed using the density operator formalism and the Master equation, which will be the subject of future investigations. In particular, our simple perturbative approach restricts our solution to examine the quantum fluctuations around a stable hyperbolic fixed point. On the other hand, nonhyperbolic fixed points and nonsteady state attractors (such as limit cycles) require more sophisticated nonperturbative treatment (while their implications for correlations and sensing remain unexplored). One such treatment involves expanding the density operator in a nondiagonal coherent state basis (so-called positive P representation), deriving a Fokker–Planck equivalent of the Lindblad Master equation and simulating the associated stochastic dynamics [48]. However, to the best of our knowledge, Fokker–Planck equations corresponding to more than two bosonic operators [66], [69] have not been well studied; our nonlinear multiresonant gyroscope is described by 4 coupled Langevin equations and will lead to an 8 + 1 dimensional Fokker Planck equation, which requires substantial computational resources and will be the subject of future investigations.
– In our approach, we have assumed idealized sources and detectors in order to simplify our gyroscopic model to physically most crucial components and thereby to unveil the fundamental information-theoretic limits (in the same spirit as the analysis presented in Ref. [42] or Section 4 of Ref. [7]). Future works will develop more detailed models that can compute commonly accepted experimental metrics such as the integration-time dependent Allan deviation curve [70], for example, by incorporating the quantum theory of photodetection [71], [72], which can explicitly take into account photo-electron generation rates and detector integration times.
– Last but not least, we note that our present model focuses on *χ* ^(2)^ processes to delineate their effects on the gyroscopic sensitivity. A more thorough gyro model may also consider *χ* ^(3)^ (Kerr-type self-modulation) nonlinearities, which may come into effect at ultra-high quality factors and are found to limit the sensitivity of the (otherwise) *linear* gyroscope [7], [16]. While we shall take into account *χ* ^(3)^ processes in detailed comprehensive models in the future (see Section 5), we note that Eqs. (16)–(19) are fully applicable to material platforms, such as thin film lithium niobate [56], [73], which possess prominent *χ* ^(2)^. Furthermore, unlike their linear counterparts, resonators with *χ* ^(2)^ can be engineered to exhibit negative Kerr shifts via cascaded second-order effects [74], which can mitigate the intrinsic positive Kerr shift; we shall investigate such cancellation schemes in our future works. On the other hand, we would like to emphasize that *χ* ^(3)^ processes, including even the Kerr shift, need not be treated as a nuisance, but as extra complexities and additional degrees of freedom that can be optimized to our advantage (see Section 5). For example, it has been recently reported that the bistability effects associated with the Kerr-shift self-modulation can even enhance sensitivities under appropriate sensing schemes [75], [76].

### 2.3 Thin film lithium niobate as an implementation platform

Our nonlinear multiresonant gyroscope can be implemented in any thin film material platform, including LiNbO3 [73], AlN [77], SiC [78], GaAs [79], etc., which has prominent *χ* ^(2)^. In this work, we consider thin film lithium niobate (TFLN) as a particularly promising platform, as it has gained widespread popularity for realizing quantum-grade ultra-low loss photonic integrated circuits [80], [81]. Indeed, lithium niobate has been traditionally employed in quantum optics applications as a nonlinear medium for generating squeezed light and entangled photon states [82], [83]. However, traditional LN crystals are bulky and suffer from relatively limited strength of light–matter interactions (leading to very weak nonlinear coupling *χ* ∼ 10^3^ Hz in Eqs. (21)). Only recently, high quality wafer-scale TFLN becomes widely available for realizing integrated photonic circuits with nonlinear and electro-optic functionalities [73]. Associated with large *χ* ^(2)^, low optical loss and strong nanophotonic confinement [84], TFLN devices offer orders of magnitude enhancements in nonlinear coupling *χ* ∼ 10^6^ Hz [56].

**Figure 3:**
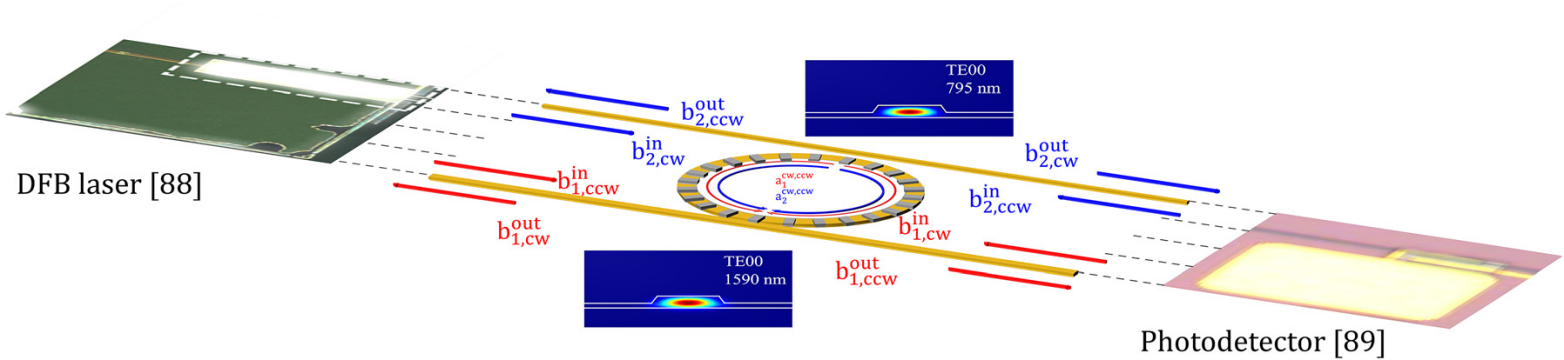
The 3D schematic (not drawn to scale) of quasi-phase matching (QPM) achieved by periodic poling. Poled rings are used to form quasi-phase matched structures. Semiconductor lasers [88] and photodetectors [89] are also integrated on the chip. The numerically simulated field profiles of both the fundamental frequency (1590 nm) and the second harmonic (795 nm) cavity modes are shown in the inset.

Figure 3 shows the design of a TFLN ring resonator used in our gyroscope. Note that feeder waveguides of different dimensions, frequency cutoffs, and dispersion characteristics can be designed to selectively couple to the fundamental (1590 nm) and the second harmonic (795 nm) modes [85], and their coupling rates can be further tuned by TFLN electro-optics [86]. To realize strong nonlinear coupling *χ* between the two resonances, two zeroth-order transverse electric eigenmodes (TE00) can be (quasi-) phase-matched [56] via periodic poling [87] that achieves crystal domain inversion, leading to periodically varying nonlinear susceptibility *χ* ^(2)^, which compensates wave vector mismatch between the fundamental and the second harmonic modes *k*_*χ*_ = *k*(*ω*_2_) − 2*k*(*ω*_1_) [56]. Apart from the phase-matched resonator itself, a fully integrated optical gyroscope can be implemented in TFLN, incorporating flip-chip bonded semiconductor lasers [88] and heterogeneously integrated uni-traveling carrier photodetectors [89]. Furthermore, we note that TFLN comes with unique electro-optic control capabilities [73] which can be used for tuning resonator parameters such as the coupling rates to the waveguides [90], managing long-term temperature stability, canceling thermal drifts and electronic noise [15], [91], and performing signal processing [92]. In our gyroscopic model, intrinsic losses *γ* and back-scattering rates *β* should be treated as “fixed” parameters, which depend on the experimentally feasible characteristics of a particular implementation platform, such as residual material losses and surface roughness due to fabrication imperfections. In thin film lithium niobate, intrinsic quality factors reaching $∼10^{8}$ have been demonstrated [93], and we expect proportionate back-scattering rates with the same order of magnitude. It is important to realize that, apart from *γ* and *β*, almost all other parameters can be designed, engineered, and optimized, including injection powers, coupling rates, resonator radius, resonator waveguide cross section and dispersion, as well as quasi-phase matching processes. We will utilize these parameters as degrees of freedom (DoFs) in optimizing the Fisher Information and, hence, the minimum detectable rotation (MDR) of our nonlinear multiresonant gyroscope. While any optimization algorithm can be employed, gradient-free global optimization methods are most suitable for a relatively low-dimensional problem like our two-resonance gyro (where about 5–10 DoFs can be optimized). We will use Bayesian optimization, a simple but powerful machine-learning–based optimization algorithm, which requires relatively few function evaluations (as compared to other heuristic methods such as simulated annealing and evolutionary algorithms [94], [95]) and has been observed to be particularly effective for optimizing ∼20 DoFs [44].

## 3 Results

Using Bayesian optimization algorithm, we holistically maximize the nonlinear multiresonant Fisher Information with respect to injection powers and coupling *Q*’s. In our optimized designs, the operational wavelengths (of the input/output light) are fixed at *λ*_1_ = 1590 nm and at *λ*_2_ = 795 nm. The material property of TFLN is taken from the literature [84]: the refractive index is *n* = 2.2 while the second-order nonlinear susceptibility is *χ* ^(2)^ = 30 pm/V. The intrinsic quality factors are fixed at *Q*_*i*1_ = 10^7^ for the 1590 nm and *Q*_*i*2_ = 10^6^ for the 795 nm. The back-scattering rates for both cavity modes are assumed to be *β*_1_ = 5.4 × 10^4^ Hz and *β*_2_ = 5.4 × 10^5^ Hz; these values are inferred by adjusting the literature-reported values [16] to the intrinsic quality factors *Q*_*i*_ of our TFLN platform. We have also fixed the radius of the resonator at *R* = 20 mm as well as the cross-sectional dimensions of the resonator waveguide (1.2 μm width × 0.6 μm thickness) and the fabrication side wall angle of 75°, leading to a cross-sectional area of 0.8 μm^2^. The two TE00 modes have phase mismatch of 1.354 μm^−1^, which can be compensated by poling with a period of 4.64 μm. Based on Eq. (21), the quasi-phase matched *χ* ^(2)^ [56], and the numerically simulated modal overlapping factor *ζ* = 1.18/μm, we calculated the nonlinear coupling strength *χ* = 1.26 × 10^6^ Hz, which is independent of the injection schemes. The rest of the parameters, including the injection powers *P*_1_ and *P*_2_ at the fundamental and the second harmonics as well as the quality factors *Q*_*c*1_ and *Q*_*c*2_ due to waveguide couplings, are to be determined by Bayesian optimization. We will investigate gyroscopic performance under different injection schemes including (1) coherent state input at the second harmonic (*λ*_2_), (2) coherent state input at the subharmonic (*λ*_1_), and (3) coherent state inputs at both second and subharmonics (*λ*_1_ and *λ*_2_).

### 3.1 Optical parametric oscillator gyro (coherent injection at second harmonic)

**Figure 4:**
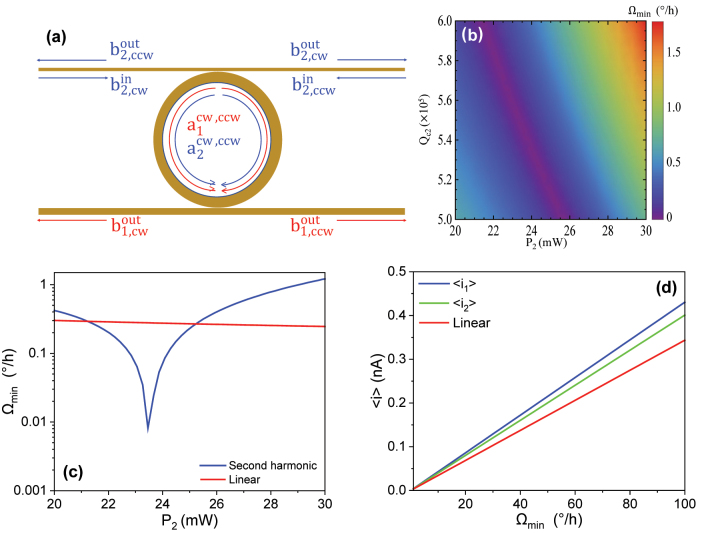
The second harmonic injection scheme. (a) The schematic of the second harmonic injection scheme. The input light is only injected at the second harmonic (blue arrow). (b) 2D density plot of the sensitivity at second harmonic injection. The sensitivity (MDR) is expressed as a function of the input power *P*_2_ and the quality factor due to coupling loss of the second harmonic cavity mode *Q*_*c*2_. (c) 1D plot of the sensitivity of the second harmonic injection scheme (blue) and the standard linear gyroscope (red) at optimal *Q*_*c*_ in terms of the input power. (d) 1D linear plot of the mean differential current of the output light at fundamental frequency (blue), second harmonic (green), and the standard linear gyroscope (red).

First, we study the performance of an optical parametric oscillator gyroscope under the coherent injection at the second harmonic frequency. As shown in Figure 4(a), classical laser light with a wavelength *λ*_2_ = 795 nm is injected from opposite directions from the waveguide ports $b_{2\text{,cw}}^{\text{in}}$ and $b_{2\text{,ccw}}^{\text{in}}$ , while no light is injected from the waveguide port $b_{1\text{,cw}}^{\text{in}}$ or $b_{1\text{,cw}}^{\text{in}}$ , i.e., *P*_1_ = 0. The carefully phase-matched fundamental and second harmonic modes facilitate parametric down conversion, in which one photon with higher frequency (shorter wavelength *λ*_2_) breaks down into two photons with half the frequency (longer wavelength *λ*_1_ = 2*λ*_2_) [48]. Using Bayesian optimization, we identify a high sensitivity regime in the 3D parameter space (*P*_2_, *Q*_*c*1_, *Q*_*c*2_). Figure 4(b) shows a 2D density plot of a small-MDR, high-sensitivity regime (0–1.7°/h), as a function of *P*_2_ and *Q*_*c*2_. Note that the optimization only considers *P*_2_ > 14.05 mW, a power threshold below which the steady-state solutions of the system become unstable [58]. In particular, the steady states of the OPO gyro are obtained from a set of nonlinear dynamical equations described in Ref. [58]. At second harmonic injection, the threshold power is given by $P_{c}=\frac{\hbar \omega_{2}{\left(\kappa_{1}+\gamma_{1}\right)}^{2}{\left(\kappa_{2}+\gamma_{2}\right)}^{2}}{16\kappa_{2}\chi^{2}}=14.05$ mW. This is the power threshold below which there only exists zero subharmonic field solution and above which two stable solutions with nonzero subharmonic fields exist. This threshold also marks the onset of parametric oscillations. High sensitivity is observed over a sizable parameter range, not at an isolated singularity. To elucidate the sensitivity enhancement, Figure 4(c) compares the MDR of our OPO gyro (solid blue line) against that of a standard linear gyro (solid red line) with the same footprint and intrinsic quality factor. The optimal coupling factors for the OPO gyro are *Q*_*c*1_ = 1.018 × 10^5^ and *Q*_*c*2_ = 5.462 × 10^5^, both discovered by Bayesian optimization. Meanwhile, the performance of the linear gyro is optimized at *Q*_*c*1_ = 9.58 × 10^6^, which is close but not exactly equal to *Q*_*i*1_ = 10^7^, considering the influence of the small but nonzero back-scattering (see Eq. (14)). As the input power is increased from 20 mW to 30 mW, the MDR of the OPO gyro drops from ∼0.42°/h down to near zero and rises back to ∼1.21°/h with a local minimum ∼0.004° /h at the *optimal* power of *P*_2*c*_ = 23.507 mW, corresponding to an optimal sensitivity point. Meanwhile, the MDR of the linear gyroscope remains >0.245° /h. Therefore, at the optimal sensitivity point, the OPO gyro is ∼ 62.3× more sensitive than the linear gyro for the same injection power, resonator size, and intrinsic quality factors. We also plotted the mean current values as a function of the rotation rate Ω at different frequency outputs in Figure 4(d), showing that the output signals of our nonlinear gyro has a stronger dependence on the rotation rate than that of the linear gyro.

### 3.2 Coherent injection at the fundamental frequency

**Figure 5:**
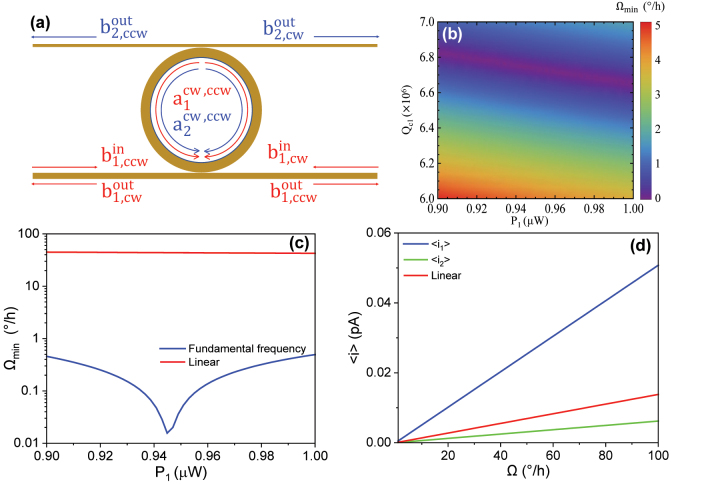
The fundamental frequency injection scheme. (a) The schematic of the fundamental frequency (subharmonic) injection scheme. The input light is only injected at the fundamental frequency (red arrow). (b) 2D density plot of the sensitivity at the fundamental frequency injection scheme. The sensitivity (MDR) is expressed as a function of the input power *P*_1_ and the quality factor due to coupling loss of the cavity mode *Q*_*c*1_. (c) 1D plot of the sensitivity of the fundamental frequency injection scheme (blue) and the standard linear gyroscope (red) at optimal *Q*_*c*_ in terms of the input power. The inset shows the magnified plot of the critical region. (d) 1D linear plot of the mean differential current of the output light at fundamental frequency (blue), second harmonic (green), and the standard linear gyroscope (red).

We also investigated the gyroscopic performance of our nonlinear resonator under a fundamental frequency injection scheme. As shown in Figure 5(a), light at input wavelength *λ*_1_ = 1590 nm is injected from the waveguide ports $b_{1\text{,ccw}}^{\text{in}}$ and $b_{1\text{,cw}}^{\text{in}}$ while *P*_2_ = 0, stimulating intracavity up-conversion (two photons of lower energy are combined to one photon of higher energy). The density plot Figure 5(b) shows a low MDR regime in terms of *P*_1_ and *Q*_*c*1_. Note that the steady-state solutions of the cavity modes are stable only when *P*_1_ < 3.24 mW [58]. Via Bayesian optimization, the optimal sensitivity point (MDR ∼0.093° /h) is found at *P*_1_ = 0.945 μW, *Q*_*c*1_ = 6.747 × 10^6^, *Q*_*c*2_ = 6.675 × 10^7^, while the MDR of the linear gyro remains elevated at ∼44°/h, leading to ∼471.3× improvement in sensitivity over a linear gyro with the same power, footprint, and intrinsic *Q*. Importantly, the fundamental frequency injection scheme merges high sensitivity and low power consumption together in a compact form, which shows great potential in practical applications.

## 4 Discussion

Table 1 summarizes the maximal sensitivity enhancement factors (over the linear baseline) that can be obtained in multiple operational regimes over a wide range of critical power requirements. Under the optimal second harmonic injection at ≈23.5 mW, our nonlinear multiresonant gyroscope can be nearly 62.3× more sensitive than an optimized linear gyro with the same radius, intrinsic quality factor, and power budget, allowing for a minimum detectable rotation (MDR) as small as 0.0044°/h. Alternatively, even larger enhancement factors (470×) can be obtained at lower powers under the fundamental injection scheme. It is important to note that, in either of the fundamental or the second harmonic injection scheme, we measure the output signals at both frequencies in order to fully utilize the input pump power (which gets converted into both harmonics), setting up a fair comparison to a linear gyro under the same pump power. For a more conservative comparison, one may argue for using dual inputs and outputs in the linear case. Aside from the fact that having to use two different frequency lasers can be disadvantageous, a simple calculation readily shows that measuring two *noninteracting* resonances in a linear gyro can offer only up to $\sqrt{2}\times$ improvement (under the same power budgets) – in fact, much less than $\sqrt{2}$ due to the smaller *Q*_*i*2_ – highlighting that nonlinear effects are indeed indispensable for significant sensitivity enhancements. Most importantly, the crucial insight we have drawn from our investigations is to realize that multiple resonances in a nonlinear resonator can be engineered to reinforce each other through nonlinear wave mixing and can be used as powerful degrees of freedom to optimize sensitivities. This critical realization suggests an exciting future direction: to generalize our gyroscope design from just two resonances to many more nonlinearly interacting resonances (see also Section 5), which may lead to even better sensitivities and functionalities (approaching the ultimate Heisenberg limit). We may further dissect the sensitivity enhancement by analyzing the multiresonant Fisher Information (Eq. (27)), which can be expanded to three terms:

(30) $$\begin{array}{ll} I\left(\delta \right) & =I_{1}+I_{2}+I_{12}\\ & =\frac{{\left(\frac{d〈{\hat{i}}_{1}〉}{d\delta }\right)}^{2}}{\Delta_{1}^{2}-\frac{\Delta_{12}^{2}}{\Delta_{2}^{2}}}+\frac{{\left(\frac{d〈{\hat{i}}_{2}〉}{d\delta }\right)}^{2}}{\Delta_{2}^{2}-\frac{\Delta_{12}^{2}}{\Delta_{1}^{2}}}+\frac{2\left(\frac{d〈{\hat{i}}_{1}〉}{d\delta }\right)\left(\frac{d〈{\hat{i}}_{2}〉}{d\delta }\right)}{\Delta_{12}-\frac{\Delta_{1}^{2}\Delta_{2}^{2}}{\Delta_{12}}} \end{array}$$

Each of these terms may be compared with the Fisher Information of the linear gyro:

(31) $$I_{L}=\frac{{\left(\frac{d\left\langle {\hat{i}}_{L}\right\rangle }{d\delta }\right)}^{2}}{\Delta_{L}^{2}}$$

Here, $\Delta_{n}^{2}=\left\langle {\hat{i}}_{n}^{2}\right\rangle -{\left\langle {\hat{i}}_{n}\right\rangle }^{2}$ and $\Delta_{12}=\frac{\left\langle {\hat{i}}_{1}{\hat{i}}_{2}\right\rangle +\left\langle {\hat{i}}_{2}{\hat{i}}_{1}\right\rangle }{2}-\left\langle {\hat{i}}_{2}\right\rangle \left\langle {\hat{i}}_{1}\right\rangle$ are the variances and the covariance of the output differential currents. First, it is immediately obvious that the Fisher Information of each injection scheme is dominated by either *I*_1_ or *I*_2_, as shown in Table 2. To better understand these terms, we further analyze the key quantities, the mean current sensitivities $\frac{d\left\langle {\hat{i}}_{n}\right\rangle }{d\delta }$ , and the variances, against their linear counterparts, in Table 3. For the second harmonic injection, while the mean current sensitivities are enhanced compared to the linear counterpart, the main improvement in *I*_2_ surprisingly comes from the noise reduction in $\Delta_{2}^{2}$ in the second harmonic differential current (relative to $\Delta_{L}^{2}$ ). This reduction results from a 7.14 dB squeezing in the second harmonic mode ${\hat{b}}_{2}^{\text{out}}$ (as opposed to the more typical squeezing in the down-converted fundamental mode in the *weakly nonlinear* regime [50]) as well as from additional noise cancellations occurring in a *strongly interacting nonlinear regime*.

**Table 2:** Fisher Information of various injection schemes.

	*I*	*I*_1_	*I*_2_	*I*_12_	*I*_*L*_
SHI	1,653,744	1.765	**1,653,743**	−0.266	427.3
FFI	3814.65	**3814.57**	0.000085	0.078	0.017

The bold values represent the major contributing terms to the sensitivity enhancement.

**Table 3:** Statistical properties of the output differential currents of various injection schemes and the linear gyroscope.

	${\left(\frac{\frac{\partial \left\langle \hat{i_{1}}\right\rangle }{\partial \delta }}{\frac{\partial \left\langle \hat{i_{L}}\right\rangle }{\partial \delta }}\right)}^{2}$	${\left(\frac{\frac{\partial \left\langle \hat{i_{2}}\right\rangle }{\partial \delta }}{\frac{\partial \left\langle \hat{i_{L}}\right\rangle }{\partial \delta }}\right)}^{2}$	$\frac{{\Delta_{1}}^{2}}{{\Delta_{L}}^{2}}$	$\frac{{\Delta_{2}}^{2}}{{\Delta_{L}}^{2}}$	$\frac{{\Delta_{12}}^{2}}{{\Delta_{1}}^{2}{\Delta_{L}}^{2}}$	$\frac{{\Delta_{12}}^{2}}{{\Delta_{2}}^{2}{\Delta_{L}}^{2}}$
SHI	1.153	0.343	278.61	8.79 × 10^−5^	5.34 × 10^−13^	1.69 × 10^−6^
FFI	13.53	0.025	6.12 × 10^−5^	5.04	0.023	2.89 × 10^−7^

**Figure 6:**
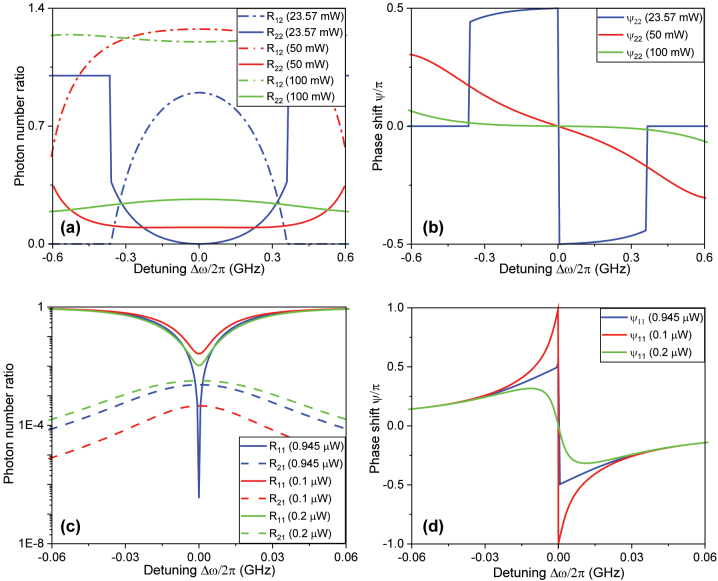
The photon number ratios ((a) and (c)) and the phase shifts ((b) and (d)) between the input/output light at all three injection schemes as a function of the detuning between the cavity resonant frequency and laser frequency. *R*_*ij*_ and *ψ*_*ij*_ are the photon number ratio and the phase shift between the input (j) and the output (i) frequencies, where 1 stands for the fundamental frequency and 2 stands for the second harmonic.

The fact that the device is operating in a strong nonlinear regime is evidenced by the observation that at the critical optimal power, the second harmonic input is *almost* completely depleted to down conversion and radiation, Figure 6(a). Note that the emergence of *nonlinear critical coupling points* characterized by complete depletion of injected power due to maximal frequency conversion has been studied previously [96], [97], [98], [99], [100], [101], [104]. Curiously, optimization of the Fisher Information reveals that a nonlinear multiresonant gyroscope also operates optimally *in the vicinity of* such a point. In the case of fundamental injection, both mean current sensitivity $\frac{d\left\langle {\hat{i}}_{1}\right\rangle }{d\delta }$ and the noise reduction in $\Delta_{1}^{2}$ significantly contribute to the enhancement of *I*_1_. We found that the noise reduction in $\Delta_{1}^{2}$ results from 4.8 dB squeezing in the fundamental mode combined with additional nonlinear cancellations, again indicating a strongly interacting nonlinear regime. More remarkably, we observe that the larger improvement in $\frac{d\left\langle {\hat{i}}_{1}\right\rangle }{d\delta }$ stems from the more sensitive nonlinear critical coupling with sharper detuning transitions at the optimal injection power (Figure 6(c) and (d)).

In our doubly resonant gyro, part of the noise reduction, observed in the strongly interacting nonlinear regime, may be more intuitively understood by contrasting with the noise characteristics of the linear gyro. In fact, it is easy to show that the differential current noise may vanish, $\Delta_{L}^{2}\to 0$ , *even in a linear gyro* at critical coupling (*κ* = *γ*) in the limit of zero back-scatting (*β* → 0), characterized by the destructive interference between the out-going cavity field $\sqrt{\kappa }\alpha$ and the incoming waveguide field ${\hat{b}}^{\text{in}}$ [102], [103]. Of course, in this limit, the mean current derivative also vanishes, $\frac{d\left\langle {\hat{i}}_{L}\right\rangle }{d\delta }\to 0$ , thus keeping the ultimate inferential sensitivity finite, i.e., yielding the shot-noise limit $∼\gamma /\sqrt{P}$ . On the other hand, a finite back-scattering *β* ≠ 0 spoils the critical coupling condition in a linear gyro, because a nonzero *β* effectively detunes the cavity resonances by lifting the degeneracy between the cw and the ccw modes, leading to an elevated noise variance when *β* is nonzero. Such a deleterious effect of *β* is mitigated in the nonlinear gyro by virtue of injected power, which can effectively “re-tune” the resonances (through the nonlinear coupling terms) and partially restore the critical coupling condition, thereby reducing the noise variance. On the other hand, this partially restored nonlinear critical coupling condition is tied to a critical power associated with (almost) perfect nonlinear frequency conversion [104]. Therefore, optimal noise reduction is observed at the same critical power (the local minimum in Figure 4(a) as well as in Figure 5(a)). We note that noise reductions have been reported in different nonlinear systems [96], [97], [98], [99], [100], [101]. Future works will pursue an in-depth comparison and understanding of our results in the context of prior reports on nonlinear noise cancellations.

## 5 Summary and outlook

We have introduced a new type of nonlinear multiresonant quantum photonic gyroscope that simultaneously achieves high sensitivity, high compactness, and low power consumption. Our analysis shows that, the gyroscope sensitivity enhancement stems from a multitude of effects, including enhanced mean current sensitivity, noise squeezing, and nonlinear noise cancellations. Specifically, we analyzed and optimized the gyroscope sensitivity of a doubly resonant *χ* ^(2)^ cavity, revealing that, under quantum noise conditions, ≳470× enhancement is possible over the classical shot noise limit. A maximum sensitivity of 0.0044°/h has been achieved at an input power of 23.507 mW at the second harmonic injection, exhibiting comparable sensitivity performance and much lower power consumption than state-of-the-art FOGs (Boreas D90: 0.001°/h, 12 W) and RLGs (Honeywell GG1320AN: 0.0035°/h, 1.6 W) [105], [106]. In addition, our design operates under classical laser injection (which can be integrated on the same chip as the gyro) and does not require complex external quantum light sources. We highlight that our current design, which uses two resonances, represents only an elementary step and a relatively simple example. In future works, we will develop a *comprehensive* inertial sensing paradigm, where a synergistic amalgamation of both quadratic *χ* ^(2)^ and cubic *χ* ^(3)^ nonlinearities, along with multiple intermixing resonances, mutually reinforced Sagnac shifts, co-arising noise squeezing and cancellations, electro-optics dynamical control and geometrically induced anomalous dispersion effects, can unleash extraordinary complexities and freedoms, which can be fully exploited by state-of-the-art optimization techniques [107], [108], [109], [110] in order to identify unprecedented regimes for gyroscopic operation and sensitivities. A full incarnation of our gyroscope design can be described by a Heisenberg–Langevin system of the form (or an equivalent density-operator Master equation [66]):

(32) $$\begin{array}{ll} \frac{d{\hat{a}}_{j}^{\mu }}{dt} & =\left(i\omega_{j}+i\delta_{j}^{\mu }\left(\Omega \right)-\frac{\kappa_{j}}{2}-\frac{\gamma_{j}}{2}\right){\hat{a}}_{j}^{\mu }+i\beta_{j}{\hat{a}}_{j}^{\nu }\\ & \;+\underset{kl\alpha \beta }{\sum }f_{kl\alpha \beta }^{\left(2\right)}\left({\hat{a}}_{k}^{\alpha },{\hat{a}}_{l}^{\beta },{\left({\hat{a}}_{k}^{\alpha }\right)}^{†},{\left({\hat{a}}_{l}^{\beta }\right)}^{†}\right)+\underset{klm\alpha \beta \theta }{\sum }\\ & \;\times f_{klm\alpha \beta \theta }^{\left(3\right)}\left({\hat{a}}_{k}^{\alpha },{\hat{a}}_{l}^{\beta },{\hat{a}}_{m}^{\theta },{\left({\hat{a}}_{k}^{\alpha }\right)}^{†},{\left({\hat{a}}_{l}^{\beta }\right)}^{†},{\left({\hat{a}}_{m}^{\theta }\right)}^{†}\right)\\ & \;+\underset{k}{\sum }\sqrt{\kappa_{jk}^{c}}{\hat{a}}_{jk,in}^{\mu }+\underset{k}{\sum }\sqrt{\gamma_{jk}^{r}}{\hat{\eta }}_{jk}^{\mu } \end{array}$$

for a selected set of carefully phase-matched and dispersion-engineered resonances {*ω*_*j*_, *j* = 1, …, *N*}. Here, *δ* ^*μ*^(Ω), *μ* ∈ {cw,ccw}, is the rotation-dependent Sagnac shift in the CW or CCW mode at each resonance. The functions *f* ^(2)^ and *f* ^(3)^ are polynomials of the annihilation and creation operators, representing all possible three-wave mixing and four-wave mixing interactions between the selected resonances; these processes include sum and difference frequency generations of different orders and combinations as well as Kerr-variety self-phase and cross-phase modulation, and even cascaded processes [111]. It is important to note that the strengths of different *f* ^(2)^ and *f* ^(3)^ terms are determined by nonlinear coupling factors [57], which characterize the field concentration and nonlinear overlaps of the modes of the photonic resonator and can be computed from nanophotonic simulations. Therefore, on-chip structural parameters, ranging from a few simple shape parameters to entire permittivity distributions, can serve as design degrees of freedom [112], by which we can engineer and optimize the different nonlinear processes (e.g., their relative contributions). The outputs of this multiresonance system are collected by multiple waveguide ports and are set to passively interfere with each other and/or go through active electro-optics pulse processing (readily achievable on a TFLN platform [73]) before arriving at multiple photodetectors to yield multiple photocurrent signals $i=\left\{{\hat{i}}_{1},\ldots ,{\hat{i}}_{M}\right\}$ . From these multi-variable (vector-valued) measurements, one can perform deep inferential analysis (such as advanced Bayesian computing [113]) to deduce the underlying noninertial motion; the sensitivity of the entire process can be characterized by an end-to-end computation of Fisher Information, which will serve as an optimization figure of merit. We recognize tremendous opportunity in analyzing and optimizing such a system with increasing levels of mathematical and computational vigor, starting from steady-state analysis, small-signal modeling, classical stochastic simulations, to the nonperturbative phase-space apparatus involving positive P-representations, Fokker–Planck equations, and stochastic calculus [48], [66], from few-parameter deterministic global optimization [108], [114], multi-parameter Bayesian optimization [44] and evolutionary algorithms [115], machine-learning assisted hybrid optimization [116], and Monte Carlo gradient computations [110] to billion-voxel topology optimization [117], inverse design by adjoint optimization [118], and full end-to-end inverse design [119] of the entire workflow from the underlying resonator geometry to multi-variable inferential processes. Experimentally, thin film lithium niobate (TFLN) continues to offer the most suitable platform, which features state-of-the-art on-chip frequency combs, pulse shaping, frequency shifting, and ultra-fast signal processing capabilities [81], [120], [121], [122].

## Supplementary Material

The online version offers supplementary material on the details of the derivations of the steady state solutions of the nonlinear coupled equations and the algebra of the quantum operators.

## Supplementary Material

Supplementary Material Details
