# Supplementary material for: Bayesian optimization of Fisher Information in nonlinear multiresonant quantum photonics gyroscopes
Source: Nanophotonics. 2024 Mar 22;13(13):2401–16. doi: 10.1515/nanoph-2024-0032 (PMC11501923; doi:10.1515/nanoph-2024-0032)
Supplement: Supplementary file 1 — Supplementary Material Details [file j_nanoph-2024-0032_suppl_001.pdf]

Research Article

Mengdi Sun*, Vassilios Kovanis, Marko Lončar, and Zin Lin

# Supplementary material: Bayesian Optimization of Fisher Information in Nonlinear Multi-Resonant Quantum Photonics Gyroscopes

## 1 Steady state solutions

As discussed in Section II B, the nonlinear coupled equations are solved by linearization. The classical scalar valued amplitudes $\alpha$ are obtained from the steady state analysis of Eqs. 14-17. To evaluate the steady state solutions, the noise terms containing the quantum operators are omitted. The equations are thus simplified as follows:

$$f_1 = \left(\frac{\kappa_1}{2} + \frac{\gamma_1}{2} - i\delta_1\right) a_{1,\mathrm{cw}} - i\beta_1 a_{1,\mathrm{ccw}} - \chi a^*_{1,\mathrm{cw}} a_{2,\mathrm{cw}} - \sqrt{\kappa_1} b^{\mathrm{in}}_{1,\mathrm{cw}} \quad (1)$$

$$f_2 = \left(\frac{\kappa_1}{2} + \frac{\gamma_1}{2} + i\delta_1\right) a_{1,\mathrm{ccw}} - i\beta_1 a_{1,\mathrm{cw}} - \chi a^*_{1,\mathrm{ccw}} a_{2,\mathrm{ccw}} - \sqrt{\kappa_1} b^{\mathrm{in}}_{1,\mathrm{ccw}} \quad (2)$$

$$f_3 = \left(\frac{\kappa_2}{2} + \frac{\gamma_2}{2} - i\delta_2\right) a_{2,\mathrm{cw}} - i\beta_2 a_{2,\mathrm{ccw}} + \frac{1}{2}\chi a^2_{1,\mathrm{cw}} - \sqrt{\kappa_2} b^{\mathrm{in}}_{2,\mathrm{cw}} \quad (3)$$

$$f_4 = \left(\frac{\kappa_2}{2} + \frac{\gamma_2}{2} + i\delta_2\right) a_{2,\mathrm{ccw}} - i\beta_2 a_{2,\mathrm{cw}} + \frac{1}{2}\chi a^2_{1,\mathrm{ccw}} - \sqrt{\kappa_2} b^{\mathrm{in}}_{2,\mathrm{ccw}} \quad (4)$$

Next, the steady state solutions are hence obtained by solving the equations $F(f_1, f_2, f_3, f_4) = 0$. Aside from the cavity modes $a_{n,cw/ccw}$, the input fields are also expressed as the steady states $b_{n,cw/ccw}$. These values are determined by the input power $P_n$ at both waveguide ports as discussed in Equation 3. Here we fix $b_n$ as real values, which reduces the phase noise as reported by Dowling [1]. At different injection schemes, different arrangements of $b_n$ are employed. For example, $b_2 = 0$ at when the input light is injected at the fundamental frequency and $b_1 = 0$ at the second harmonic injection. Though the steady state solutions of a similar system has been studied by Drummond [2], in which the analytical solutions are given at each injection scheme. In our system, however, the rotation-induced frequency shift $\delta_n$ and the Rayleigh back-scattering $\beta_n$ which introduces cross-coupling between the CW and the CCW modes make it impossible to calculate the analytical solution, hence we calculated the numerical solutions instead. Since these equations are nonlinear equations, multiple solutions are expected at each set of parameters. In order to discover the steady state solutions, linear stability analysis is performed [3, 4]. By checking the eigen values of the Jacobian matrix [5] associated with each set of solution we can determine the stability of these solutions. The Jacobian matrix J of Eqs. 1-4 is obtained by taking the gradient over a vector of the unknown variables $a_{\mathrm{n,cw/ccw}}$:

$$J = \nabla F|_{a_{\mathrm{n,cw/ccw}}} \quad (5)$$

Note that in Equation 5 the function system $F$ and the variables $a_{\mathrm{n,cw/ccw}}$ are both vectors, hence the gradient operator $\nabla$ generates a matrix $J$ instead of a vector. When the real part of the eigen values of the matrix is negative, the solution is stable and can be used for further calculation.

## 2 The algebra of the quantum operators

The system is assumed to be quantum-limited, meaning that the shot noise is considered as the main source of noise. To this end, it is necessary to investigate the

***Corresponding author: Mengdi Sun,** Bradley Department of Electrical and Computer Engineering, Virginia Tech, Arlington, VA, USA, mengdis@vt.edu; https://orcid.org/0000-0001-5031-0443
**Vassilios Kovanis,** Bradley Department of Electrical and Computer Engineering, Virginia Tech, Arlington, VA, USA
**Marko Lončar,** John A. Paulson School of Engineering and Applied Sciences, Harvard University, Cambridge, MA, USA
**Zin Lin,** Bradley Department of Electrical and Computer Engineering, Virginia Tech, Blacksburg, VA, USA

statistical properties of the output light. As discussed in Section II B, the output light are expressed by the operators $\hat{b}^{\text{out}}_{\text{n,cw/ccw}}$. As shown in Fig. 2, the output light is measured by homodyne detection that $\hat{b}^{\text{out}}_{\text{n,cw}}$ and $\hat{b}^{\text{out}}_{\text{n,ccw}}$ are coupled with each other before being detected by two independent photodetectors:

$$\hat{b}_{1+} = \left(\hat{b}^{\text{out}}_{1,\text{cw}} e^{-i\phi_1} + i\hat{b}^{\text{out}}_{1,\text{ccw}} e^{i\phi_1}\right)/\sqrt{2} \quad (6)$$

$$\hat{b}_{1-} = \left(i\hat{b}^{\text{out}}_{1,\text{cw}} e^{-i\phi_1} + \hat{b}^{\text{out}}_{1,\text{ccw}} e^{i\phi_1}\right)/\sqrt{2} \quad (7)$$

$$\hat{b}_{2+} = \left(\hat{b}^{\text{out}}_{2,\text{cw}} e^{-i\phi_2} + i\hat{b}^{\text{out}}_{2,\text{ccw}} e^{i\phi_2}\right)/\sqrt{2} \quad (8)$$

$$\hat{b}_{2-} = \left(i\hat{b}^{\text{out}}_{2,\text{cw}} e^{-i\phi_2} + \hat{b}^{\text{out}}_{2,\text{ccw}} e^{i\phi_2}\right)/\sqrt{2} \quad (9)$$

Here $\phi_1$ and $\phi_2$ are the propagation phase shifts of the output light at the sub and the second harmonics, which can be arbitrarily selected. Here we set them to zero. The resultant differential current current is given by:

$$\hat{i}_1 = R\hbar\omega_1 \left(\hat{b}^\dagger_{1+}\hat{b}_{1+} - \hat{b}^\dagger_{1-}\hat{b}_{1-}\right) \quad (10)$$

$$\hat{i}_2 = R\hbar\omega_2 \left(\hat{b}^\dagger_{2+}\hat{b}_{2+} - \hat{b}^\dagger_{2-}\hat{b}_{2-}\right) \quad (11)$$

Here R is the responsivity of the photodetectors, set as 0.58 $A/W$ in our analysis. Setting $A_1 = R\hbar\omega_1$ and $A_2 = R\hbar\omega_2$, Eqs. 10-11 can be further simplified as:

$$\hat{i}_1 = iA_1 \left(\hat{b}^{\text{out}\dagger}_{1,\text{cw}}\hat{b}^{\text{out}}_{1,\text{ccw}} - \hat{b}^{\text{out}\dagger}_{1,\text{ccw}}\hat{b}^{\text{out}}_{1,\text{cw}}\right) \quad (12)$$

$$\hat{i}_2 = iA_2 \left(\hat{b}^{\text{out}\dagger}_{2,\text{cw}}\hat{b}^{\text{out}}_{2,\text{ccw}} - \hat{b}^{\text{out}\dagger}_{2,\text{ccw}}\hat{b}^{\text{out}}_{2,\text{cw}}\right) \quad (13)$$

Following Maleki's approach [6], the output operators are linearized as $\hat{b}^{\text{out}}_{\text{n,cw/ccw}} = b^{\text{out}}_{\text{n,cw/ccw}} + \delta\hat{b}^{\text{out}}_{\text{n,cw/ccw}}$. Here we analyze the perturbation terms, such that Eqs. 12-13 are simplified as:

$$\delta\hat{i}_1 = iA_1\big(b^{\text{out}}_{1,\text{ccw}}\delta\hat{b}^{\text{out}\dagger}_{1,\text{cw}} + b^{\text{out}*}_{1,\text{cw}}\delta\hat{b}^{\text{out}}_{1,\text{ccw}} - b^{\text{out}^*}_{1,\text{ccw}}\delta\hat{b}^{\text{out}}_{1,\text{cw}} - b^{\text{out}}_{1,\text{cw}}\delta\hat{b}^{\text{out}\dagger}_{1,\text{ccw}}\big) \quad (14)$$

$$\delta\hat{i}_2 = iA_2\big(b^{\text{out}}_{2,\text{ccw}}\delta\hat{b}^{\text{out}\dagger}_{2,\text{cw}} + b^{\text{out}*}_{2,\text{cw}}\delta\hat{b}^{\text{out}}_{2,\text{ccw}} - b^{\text{out}^*}_{2,\text{ccw}}\delta\hat{b}^{\text{out}}_{2,\text{cw}} - b^{\text{out}}_{2,\text{cw}}\delta\hat{b}^{\text{out}\dagger}_{2,\text{ccw}}\big) \quad (15)$$

Then quadrature basis expansion is performed to separate the real and the imaginary parts ($X$ and $Y$) of the operators:

$$b^{\text{out}}_{\text{n,cw/ccw}} = X^{\text{out}}_{\text{n,cw/ccw}} + iY^{\text{out}}_{\text{n,cw/ccw}} \quad (16)$$

$$b^{\text{out}*}_{\text{n,cw/ccw}} = X^{\text{out}}_{\text{n,cw/ccw}} - iY^{\text{out}}_{\text{n,cw/ccw}} \quad (17)$$

$$\delta\hat{b}^{\text{out}}_{\text{n,cw/ccw}} = \delta\hat{X}^{\text{out}}_{\text{n,cw/ccw}} + i\delta\hat{Y}^{\text{out}}_{\text{n,cw/ccw}} \quad (18)$$

$$\delta\hat{b}^{\text{out}\dagger}_{\text{n,cw/ccw}} = \delta\hat{X}^{\text{out}}_{\text{n,cw/ccw}} - i\delta\hat{Y}^{\text{out}}_{\text{n,cw/ccw}} \quad (19)$$

Hence, Eqs. 14-15 are converted to:

$$\delta\hat{i}_1 = 2A_1\big(X^{\text{out}}_{1,\text{ccw}}\delta\hat{Y}^{\text{out}}_{1,\text{cw}} - Y^{\text{out}}_{1,\text{ccw}}\delta\hat{X}^{\text{out}}_{1,\text{cw}} - X^{\text{out}}_{1,\text{cw}}\delta\hat{Y}^{\text{out}}_{1,\text{ccw}} + Y^{\text{out}}_{1,\text{cw}}\delta\hat{X}^{\text{out}}_{1,\text{ccw}}\big) \quad (20)$$

$$\delta\hat{i}_2 = 2A_2\big(X^{\text{out}}_{2,\text{ccw}}\delta\hat{Y}^{\text{out}}_{2,\text{cw}} - Y^{\text{out}}_{2,\text{ccw}}\delta\hat{X}^{\text{out}}_{2,\text{cw}} - X^{\text{out}}_{2,\text{cw}}\delta\hat{Y}^{\text{out}}_{2,\text{ccw}} + Y^{\text{out}}_{2,\text{cw}}\delta\hat{X}^{\text{out}}_{2,\text{ccw}}\big) \quad (21)$$

Next we need to determine the statistical properties of $\delta\hat{i}_1$ and $\delta\hat{i}_2$. Nevertheless, the output light is in complex quantum states (squeezed vacuum/squeezed coherent) which are difficult to calculate. On the other hand, these quadrature operators are nothing but linear combinations of the input light which is in relatively simple quantum states (vacuum/coherent). To this end, we calculate the mean values and the variances from the input states. Rewrite Eqs. 20 and 21 in the form below:

$$\delta\hat{i}_1 = \sum_{n=1}^{2}\big(b^{(1)}_{\text{x,n,cw/ccw}}\hat{b}^{\text{in}}_{\text{X,n,cw/ccw}} + b^{(1)}_{\text{y,n,cw/ccw}}\hat{b}^{\text{in}}_{\text{Y,n,cw/ccw}} + c^{(1)}_{\text{x,n,cw/ccw}}\hat{c}^{\text{in}}_{\text{X,n,cw/ccw}} + c^{(1)}_{\text{y,n,cw/ccw}}\hat{c}^{\text{in}}_{\text{Y,n,cw/ccw}}\big) \quad (22)$$

$$\delta\hat{i}_2 = \sum_{n=1}^{2}\big(b^{(2)}_{\text{x,n,cw/ccw}}\hat{b}^{\text{in}}_{\text{X,n,cw/ccw}} + b^{(2)}_{\text{y,n,cw/ccw}}\hat{b}^{\text{in}}_{\text{Y,n,cw/ccw}} + c^{(2)}_{\text{x,n,cw/ccw}}\hat{c}^{\text{in}}_{\text{X,n,cw/ccw}} + c^{(2)}_{\text{y,n,cw/ccw}}\hat{c}^{\text{in}}_{\text{Y,n,cw/ccw}}\big) \quad (23)$$

In Eqs. 22-23, $\hat{b}^{\text{in}}_{\text{X,n,cw/ccw}}$, $\hat{b}^{\text{in}}_{\text{Y,n,cw/ccw}}$, $\hat{c}^{\text{in}}_{\text{X,n,cw/ccw}}$ and $\hat{c}^{\text{in}}_{\text{Y,n,cw/ccw}}$ are the quadrature operators (real and imaginary parts) of the injection light $\hat{b}^{\text{in}}_{\text{n,cw/ccw}}$ and the intrinsic loss channels $\hat{c}^{\text{in}}_{\text{n,cw/ccw}}$ for sub/second (n=1,2) harmonic light, where $\hat{b}^{\text{in}}_{\text{X,n,cw/ccw}}$ and $\hat{b}^{\text{in}}_{\text{Y,n,cw/ccw}}$ are in coherent states and $\hat{c}^{\text{in}}_{\text{X,n,cw/ccw}}$ and $\hat{c}^{\text{in}}_{\text{Y,n,cw/ccw}}$ are in vacuum states. $b^{(1,2)}_{\text{x,n,cw/ccw}}$, $b^{(1,2)}_{\text{y,n,cw/ccw}}$, $c^{(1,2)}_{\text{x,n,cw/ccw}}$ and $c^{(1,2)}_{\text{y,n,cw/ccw}}$ are the corresponding coefficients of these operators at either sub or second harmonic. Assume $\psi_1/\psi_2$ and $N_1/N_2$ are the initial phases and the numbers of the injected photons of the input light $\hat{b}_{1,\text{cw/ccw}}$ and $\hat{b}_{2,\text{cw/ccw}}$, where $N_1 = |b^{\text{in}}_1|^2$ and $N_2 = |b^{\text{in}}_2|^2$. With all these ingredients, now we can calculate mean values and the variances of both differential currents. For a coherent state $|\alpha>$, the mean values of both quadra-

ture operators and their squares are given by [7]:

$$< \alpha|\hat{X}|\alpha >= \sqrt{N}\cos\psi \tag{24}$$

$$< \alpha|\hat{Y}|\alpha >= \sqrt{N}\sin\psi \tag{25}$$

$$< \alpha|\hat{X}^2|\alpha >= \frac{4N(\cos\psi)^2+1}{4} \tag{26}$$

$$< \alpha|\hat{Y}^2|\alpha >= \frac{4N(\sin\psi)^2+1}{4} \tag{27}$$

and the variances are defined as the mean values of the square minus the square of the mean values of the quadrature operators:

$$\langle\alpha|\Delta\hat{X}^2|\alpha\rangle = \langle\alpha|\hat{X}^2|\alpha\rangle - (\langle\alpha|\hat{X}|\alpha\rangle)^2 = \frac{1}{4} \tag{28}$$

$$\langle\alpha|\Delta\hat{Y}^2|\alpha\rangle = \langle\alpha|\hat{Y}^2|\alpha\rangle - (\langle\alpha|\hat{Y}|\alpha\rangle)^2 = \frac{1}{4} \tag{29}$$

Note that when two quadrature operators do not share the same eigen vectors, the definitions are different. For example, the inner product of a real quadrature operator at the second harmonic and an imaginary quadrature operator at the fundamental frequency injection is given by:

$$< \alpha|\hat{X}_2\hat{Y}_1|\alpha >= \sqrt{N_1}\sqrt{N_2}\cos\psi_2\sin\psi_1 \tag{30}$$

$$< \alpha|\hat{X}_2\hat{Y}_1|\alpha > - < \alpha|\hat{X}_2|\alpha >< \alpha|\hat{Y}_1|\alpha >= 0 \tag{31}$$

Now we can obtain the mean values and the variances of $\delta\hat{i}_1$ and $\delta\hat{i}_2$:

$$< \delta\hat{i}_1 >= \sum_{n=1}^{2}(b^{(1)}_{\mathrm{x,n,cw/ccw}}\sqrt{N_n}\cos\psi_n + b^{(1)}_{\mathrm{y,n,cw/ccw}}\sqrt{N_n}\sin\psi_n) \tag{32}$$

$$< \delta\hat{i}_2 >= \sum_{n=1}^{2}(b^{(2)}_{\mathrm{x,n,cw/ccw}}\sqrt{N_n}\cos\psi_n + b^{(2)}_{\mathrm{y,n,cw/ccw}}\sqrt{N_n}\sin\psi_n) \tag{33}$$

$$< \Delta\delta\hat{i}_1^2 > = \frac{1}{4}\sum_{n=1}^{2}([(b^{(1)}_{\mathrm{x,n,cw/ccw}})^2 + (b^{(1)}_{\mathrm{y,n,cw/ccw}})^2 + (c^{(1)}_{\mathrm{x,n,cw/ccw}})^2 + (c^{(1)}_{\mathrm{y,n,cw/ccw}})^2]) \tag{34}$$

$$< \Delta\delta\hat{i}_2^2 > = \frac{1}{4}\sum_{n=1}^{2}([(b^{(2)}_{\mathrm{x,n,cw/ccw}})^2 + (b^{(2)}_{\mathrm{y,n,cw/ccw}})^2 + (c^{(2)}_{\mathrm{x,n,cw/ccw}})^2 + (c^{(2)}_{\mathrm{y,n,cw/ccw}})^2]) \tag{35}$$

As discussed in Section II B, in order to determine the covariance matrix, it is also necessary to calculate the correlation between $\delta\hat{i}_1$ and $\delta\hat{i}_2$ following the rule of operator calculation defined above:

$$\langle\delta\hat{i}_1\delta\hat{i}_2\rangle - \langle\delta\hat{i}_1\rangle\langle\delta\hat{i}_2\rangle = \sum_{n=1}^{2}[b^{(1)}_{\mathrm{x,n,cw/ccw}}b^{(2)}_{\mathrm{x,n,cw/ccw}} + b^{(1)}_{\mathrm{y,n,cw/ccw}}b^{(2)}_{\mathrm{y,n,cw/ccw}} + c^{(1)}_{\mathrm{x,n,cw/ccw}}c^{(2)}_{\mathrm{x,n,cw/ccw}} + c^{(1)}_{\mathrm{y,n,cw/ccw}}c^{(2)}_{\mathrm{y,n,cw/ccw}}] \tag{36}$$

$$\langle\delta\hat{i}_2\delta\hat{i}_1\rangle - \langle\delta\hat{i}_2\rangle\langle\delta\hat{i}_1\rangle = \langle\delta\hat{i}_1\delta\hat{i}_2\rangle - \langle\delta\hat{i}_1\rangle\langle\delta\hat{i}_2\rangle \tag{37}$$

With everything discussed in this section, particularly Eqs. 32-37, now we can calculate Eqs. 23-25 to determine the Fisher information and the corresponding sensitivity of the system.

## 3 The sensitivity of the optimal injection power

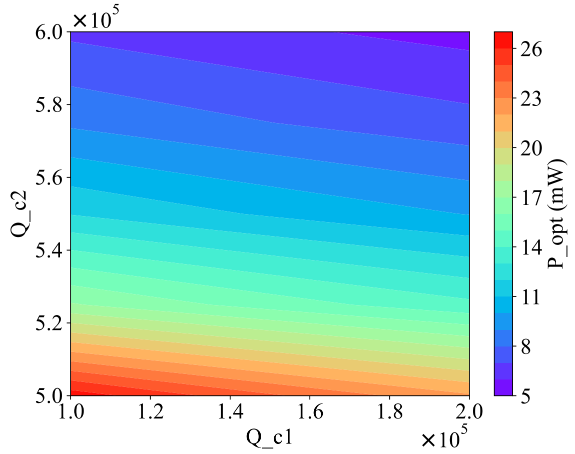


**Fig. 1:** The optimal injection power $P_{opt}$ as a function of the Q factor due to coupling losses $Q_{c1}$ and $Q_{c2}$.

## References

[1] J. P. Dowling, Correlated input-port, matter-wave interferometer: Quantum-noise limits to the atom-laser gyroscope, Phys. Rev. A 57, 4736 (1998).

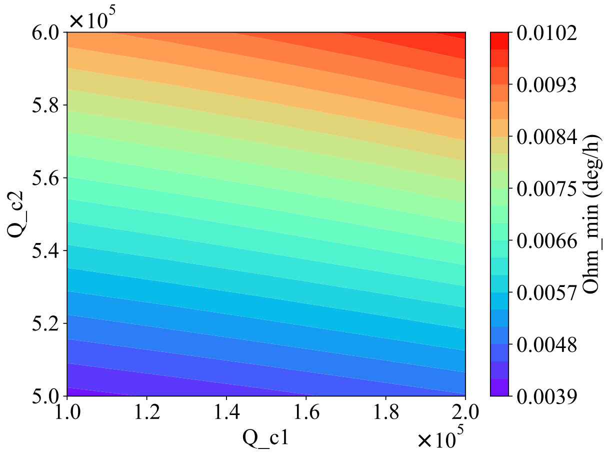


**Fig. 2:** MDR as a function of the Q factor due to coupling losses $Q_{c1}$ and $Q_{c2}$.

[2] P. Drummond, K. McNeil, and D. Walls, Non-equilibrium transitions in sub/second harmonic generation, Optica Acta: International Journal of Optics 27, 321 (1980).

[3] T. Erneux and P. Glorieux, Laser Dynamics (Cambridge University Press, 2010).

[4] A. Gavrielides, V. Kovanis, and T. Erneux, Analytical stability boundaries for a semiconductor laser subject to optical injection, Optics Communications 136, 253 (1997).

[5] H. Sayama, Introduction to the modeling and analysis of complex systems (2015).

[6] A. B. Matsko, W. Liang, A. A. Savchenkov, V. S. Ilchenko, and L. Maleki, Fundamental limitations of sensitivity of whispering gallery mode gyroscopes, Physics Letters A 382, 2289 (2018), special Issue in memory of Professor V.B. Braginsky.

[7] M. O. Scully and M. S. Zubairy, Quantum Optics (Cambridge University Press, 1997).
